# The Quality and Bioactive Properties of Mulberry Wine Under Different Fermentation Conditions

**DOI:** 10.3390/foods14193393

**Published:** 2025-09-30

**Authors:** Jiajun Li, Huiming Zhang, Tieyan Jin

**Affiliations:** College of Agriculture, Yanbian University, Yanji 133000, China; joseph4675@163.com (J.L.);

**Keywords:** mulberry wine, fermentation process optimization, volatile aroma compounds, active substance, anticancer activity

## Abstract

This study systematically investigated the effects of key fermentation parameters—initial sugar content (24–28 °Brix), temperature (15–20 °C), and yeast inoculation rate (0.04–0.12%)—on the quality, volatile aroma characteristics, antioxidant capacity, and bioactive properties of mulberry wine. Through a combination of single-factor experiments and response surface methodology (RSM), optimal fermentation conditions were determined as follows: initial sugar content of 25 °Brix, temperature of 18 °C, and yeast inoculation rate of 0.08%. Under these conditions, the resulting wine exhibited superior sensory characteristics, enhanced antioxidant activity (total phenolic content, DPPH and ABTS radical scavenging capacity, and FRAP), and significantly higher levels of key aroma compounds (e.g., ethyl acetate, phenethyl alcohol) compared to unfermented mulberry juice. Furthermore, the wine exhibited dose-dependent inhibition of proliferation in HepG2 and HT29 cells with IC_50_ values of 0.82 mg/mL and 1.05 mg/mL, respectively, and demonstrated selective antibacterial activity against Escherichia coli and Staphylococcus aureus. These findings provide a scientific basis for optimizing the production of mulberry wine with enhanced sensory qualities and functional properties, highlighting its potential as a health-promoting fermented beverage.

## 1. Introduction

Mulberry (*Fructus mori*) is the fruit of the mulberry plant (*Morus alba* L.), family Moraceae. The fruit is rich in polyphenols, volatile oils, catechins, quercetin, and vitamins, which confer antioxidant, glucose-regulating, neuroprotective, and other biological activities [1]. However, mulberries are susceptible to soft rotting and deterioration after harvest due to their high moisture content (78–85%), and the storage period at room temperature is usually less than 48 h [2]. Therefore, mulberries need to be pre-cooled (4 °C) and combined with quick-freezing processing technology for quality control, and then they can be made into lyophilized powder, a fermented beverage, or applied as a functional ingredient in nutrient-fortified food [3].

Mulberry fruit wine, a low-alcohol fermented beverage, is produced through standardized industrial processes including selection, crushing, sugar adjustment, and yeast inoculation [4]. The final product exhibits distinct characteristics, as follows: clarity, with a light transmittance exceeding 92%, a signature purple-red hue, and a complex aroma profile dominated by esters, alcohols, and terpenes, alongside notable nutritional functionality [5].

Recent advances in mulberry-based product development include a study by Ma et al. [6], who formulated a plant-based beverage with a juice-like texture by blending mulberry fruit and leaves [7]. Huang et al. [8] optimized fermentation parameters to mitigate hazardous byproducts such as fusel oils and biogenic amines. Li, Y. [9] innovated a dual-substrate co-fermentation system (mulberry/strawberry = 7:3, *v*/*v*), establishing a technical framework for high-value berry processing. Yang et al. [10] systematically mapped the dynamic evolution of physicochemical parameters (alcohol content, total acidity, and sugar levels) during fermentation and characterized aroma constituents via GC-MS [11].

Previous studies on mulberry wine have primarily focused on fundamental fermentation parameters [12], flavor components [13], and color stability [14], with few investigations comprehensively examining changes in bioactive properties throughout the entire fermentation process [15]. This study expands existing knowledge by integrating process optimization with systematic analysis of antioxidant, anticancer, and antibacterial activities. This multidimensional research approach is rarely reported in the literature. The simultaneous optimization of sensory and functional properties using a response surface methodology represents a novel contribution to the field. Furthermore, demonstrating dose-dependent cytotoxicity against cancer cell lines and antibacterial activity against specific bacteria provides new insights into mulberry wine’s health-promoting potential, positioning it as a candidate for functional beverage development. These findings not only enhance the scientific understanding of mulberry wine but also offer practical guidance for improving quality and bioactivity in industrial production.

## 2. Materials and Methods

### 2.1. Materials and Reagents

Mulberry fruit (*Morus alba* L., harvested in Shaanxi Province, China; soluble solids ≥14 °Brix), food-grade sucrose (COFCO Group, Beijing, China, compliant with GB 317-2018 standard [16], purity ≥99.5%), *Saccharomyces cerevisiae* yeast strain (Bio-Ingredients Specialist, Lavender Bay, Australia, Fermivin series; viable count ≥ 1 × 10^9^ CFU/g), composite pectinase (Bio-Ingredients Specialist, Pectinase EX-3; enzymatic activity ≥50,000 U/g), *E. coli* (ATCC 1682), *S. aureus* (ATCC 503), *S. mutans* (CGMCC 3289), human hepatocellular carcinoma cells (HepG2, SCSP-5263; Chinese Academy of Sciences Cell Bank), human colon adenocarcinoma cells (HT29, ATCC HTB-38), DMEM medium (HyClone, Logan, UT, USA, catalog no. SH30243.01; containing 4.5 g/L glucose), premium-grade fetal bovine serum (FBS; Ausbian, Greater Adelaide, Australia), MTT reagent (Beyotime Biotechnology, Haimen, China, catalog no. C0009S; 5 mg/mL tetrazolium salt), anhydrous diethyl ether (chromatographic grade, Aladdin; moisture ≤0.003%), absolute ethanol (GC-grade, Sinopharm Chemical Reagent Co., Shanghai, China, batch no. 20230617), boric acid-indicator mixed solution (Aladdin, H259748; methyl red:bromocresol green = 1:5), concentrated sulfuric acid (analytical grade), DPPH radical (1,1-diphenyl-2-picrylhydrazyl, TCI Chemicals, catalog no. D218408; purity ≥95%), 5-Fluorouracil (5-FU, MedChemExpress, HY-90006; purity 99.83%), Matrine standard (Yuanye Bio-Technology, Shanghai, China, Y-008-181021; HPLC purity ≥98%), Mueller Hinton agar (MHB, Oxoid CM0405), brain heart infusion broth (BHI, Qingdao Hope Bio-Technology, Qingdao, China, HB0128).

### 2.2. Instruments and Equipment

PHS-3C digital pH meter (Shanghai Yidian Scientific Instrument Co., Ltd., Shanghai, China; pH range: 0–14.00), ATAGO PAL-1 digital refractometer (ATAGO Co., Tokyo, Japan; Brix range: 0.0–85.0%), CM-5 spectrophotometer (Konica Minolta. Tokyo, Japan.; accuracy: ±0.2%), U-3900 dual-beam UV-Vis spectrophotometer (Hitachi, Tokyo, Japan; wavelength range: 190–900 nm), 7890B-5977B GC-MS system (Agilent Technologies, Santa Clara, CA, USA), KDN-1 automated Kjeldahl nitrogen analyzer (Dongyi Instrument Co., Ltd., Nanyang, China), HF-105A thermostatic incubator (Jiangsu Jintan Instrument Factory, Changzhou, China), Alcoholmeter (Shenyang Analytical Instrument Co., Ltd., Shenyang, China), and A51119700DPC full-wavelength microplate reader (Thermo Scientific, Waltham, MA, USA).

### 2.3. Experimental Methods

#### 2.3.1. Mulberry Wine Production Process and Key Steps

The frozen mulberries were homogenized to obtain a viscous puree, which was then treated with pectinase at a constant temperature of 50 °C for 120 min [15]. Sucrose was added to adjust the total sugar concentration to 24 °Brix, 26 °Brix, and 28 °Brix to facilitate alcoholic fermentation [17]. This brix range references common starting brix levels reported in the literature for making mulberry and similar berry fruit wines and is intended to balance yeast fermentation activity with the alcoholic and sensory balance of the final product. *Fermivin* wine yeast (inoculum size 0.08% *w*/*v*) was selected and activated in sterile water containing 5% (*w*/*v*) glucose at 38 °C for 30 min. The activated yeast culture was then introduced into the reaction system. The initial pH of the mulberry puree was approximately 4.60. It was adjusted to 4.0 ± 0.1 using citric acid, which falls within the optimal range (3.5–4.5) for *S. cerevisiae*, which effectively promotes alcoholic fermentation while inhibiting the growth of most stray bacteria (e.g., *lactic acid bacteria*, *acetic acid bacteria*). This was also performed to prevent excessive accumulation of volatile acids due to the hydrolysis of ethyl acetate during fermentation. In addition, 80 mg/L of sodium bisulfite was added to inhibit microbial growth [18]. All fermentations were carried out in 5 L glass fermenters filled with 3 L of mulberry must. The fermenters were sealed with airlocks to maintain anaerobic conditions and were not shaken or aerated during the process.

Three independent fermentation batches were performed for each experimental condition (initial sugar levels: 24 °Brix, 26 °Brix, 28 °Brix; temperatures: 15 °C, 18 °C, 20 °C). Each batch utilized freshly prepared mulberry puree and separately activated yeast cultures to ensure biological replicates.

#### 2.3.2. Effect of Initial Sugar Concentration on Wine Quality

Three initial soluble solid levels (24 °Brix, 26 °Brix, 28 °Brix) were established by sucrose supplementation. Fermentation was conducted for 15 days, as preliminary trials confirmed that both sugar consumption (%Brix) and ethanol production had plateaued by this time, indicating the completion of primary fermentation. Fermentation temperature was set at 18 °C. Physicochemical parameters (pH, residual sugar, alcohol content) and sensory attributes were monitored daily to determine the optimal initial sugar concentration.

#### 2.3.3. Effect of Fermentation Temperature on Wine Quality

Three temperature gradients (15 °C, 18 °C, 20 °C) were tested under identical substrate conditions during 15-day fermentation. Set the initial fermentation sugar content to 26 °Brix, with a yeast addition rate of 0.08%. Fermentation will continue for 15 days, during which basic physicochemical indicators (pH, residual sugar, alcohol content) and sensory characteristics will be monitored daily to determine the optimal fermentation temperature.

#### 2.3.4. Effect of Yeast Dosage on Fruit Wine Quality

By adding sucrose to fix the initial fermentation sugar content at 26 °Brix, the fermentation temperature was set to 18 °C, with yeast additions at 0.04%, 0.08%, and 0.12%. Fermentation proceeded for 15 days, with daily monitoring of fundamental physicochemical indicators (pH, residual sugar, alcohol content) and sensory characteristics to determine the optimal yeast addition rate.

#### 2.3.5. Response Surface Experiment for Fermentation Process Optimization

Using the sensory evaluation score (Y) of mulberry fruit wine as the response variable, the initial fermentation sugar content (A), fermentation temperature (B), and yeast inoculation amount (C) were selected as significant influencing factors. Response surface experiments were conducted to optimize the fermentation process, employing a three-factor, three-level Box–Behnken Design with Design-Expert 13.0 response surface design software and the yeast inoculation rate (C) as independent variables. Fermentation parameters were optimized using Design-Expert 13.0 response surface design software. A three-factor, three-level Box–Behnken Design (BBD) was implemented to analyze the influence of each factor on the response value, enabling multi-objective optimization of process parameters. Regression models were employed to examine main effects and interactions among factors, with model significance verified through analysis of variance (ANOVA) to determine the optimal values for the parameters of the mulberry fruit wine brewing process. The specific factor level table is shown in Table 1.

### 2.4. Experimental Conditions

#### 2.4.1. Total Phenolic Content Determination

Total phenolics were quantified using a modified Folin–Ciocalteu method adapted from Liu et al. [19] A standard curve was constructed with gallic acid (0.1–2.4 μg/mL). Samples and standards were reacted with 2 mL of Folin–Ciocalteu reagent and 10 mL of 10% Na_2_CO_3_, followed by 60 min incubation at 50 °C [20]. Absorbance (A) was measured at 765 nm, yielding the following linear regression equation:

$$Y=3.7366X+0.0215(R^{2}>0.995)$$

And the total phenol content was calculated according to Equation (1).

(1) $$\mathrm{Total}\;\mathrm{phenolics}\;(\mathrm{mg}\;\mathrm{GAE}/g)=\frac{V\times N\times (A-0.0215)}{3.7366\times m}$$

In Equation (1), V is the total volume of the extract (mL); N is the number of dilutions; A is the measured absorbance; and m is the sample mass (g).

#### 2.4.2. Flavonoid Content Determination

Flavonoids were analyzed via sodium nitrite–aluminum nitrate complexation [21]. A rutin standard curve (0–3.5 mg/L) was developed by sequential addition of 0.5 mL 5% *v*/*v* NaNO_2_, 0.5 mL 10% *v*/*v* Al(NO_3_)_3_, and 5 mL 4% *v*/*v* NaOH. After 30 min of incubation, absorbance at 509 nm produced the following equation [22]:

$$Y=10.061X-0.0021(R^{2}>0.996)$$

#### 2.4.3. DPPH-Clearance Measurement

Mulberry fruit wine 1.0 mL was pipetted and vortex-mixed with 2.0 mL of DPPH working solution (0.2 mmol/L), and the absorbance of the reaction system was measured at λ = 517 nm using a UV spectrophotometer after incubation in the dark for 30 min (experimental group A) [23]. In order to eliminate matrix interference, two control groups were set up in parallel: 1.0 mL of the sample solution was mixed with 2.0 mL of anhydrous ethanol (analytical purity) as a blank control, and the background value of absorbance B was measured; the other 2.0 mL of the DPPH working solution was taken with 1.0 mL of anhydrous ethanol to construct a negative control, and the baseline value of absorbance C was measured [24]. The DPPH elimination rate was calculated according to Equation (2).

(2) $$\mathrm{Clearance}\;\mathrm{rate}\;(\%)=\frac{1-(A-B)}{C}\times 100\%$$

In Equation (2), A represents the absorbance value of 1 mL of the sample solution plus 2 mL of the DPPH solution; B represents the absorbance value of 1 mL of the sample solution plus 2 mL of ethanol; and C represents the absorbance value of 1 mL of ethanol plus 2 mL of the DPPH solution.

#### 2.4.4. Determination of ABTS ^+^ Clearance

Dissolve 3 mg of ABTS in a 1.5 mL centrifuge tube, add 0.735 mL of ultrapure water to dissolve by vortexing and shaking, and prepare a 7.4 mmol/L reserve master solution (error range ± 0.5%) [25]. For synchronous preparation of the potassium persulfate initiator solution, accurately measure 1.43 mL of ultrapure water to dissolve 1.00 mg K_2_S_2_O_8_ (analytically pure) to obtain 2.6 mmol/L oxidant reserve solution. Mix at 1:1 (*v*/*v*) and incubate at room temperature away from light for 12 h. The standardized ABTS^+^ working solution was obtained by using a UV-visible spectrophotometer (pre-run for 30 min to stabilize the baseline), diluting the reaction product by 45-fold, and adjusting the absorbance value of A = 0.70 ± 0.02 at 734 nm [26]. This was calculated according to Equation (3).

(3) $$\mathrm{Clearance}\;\mathrm{rate}\;(\%)=1-\frac{A}{B}\times 100\%$$

#### 2.4.5. Analysis of Volatile Components

Volatile aroma components were analyzed using GC-MS. Chromatographic separation was performed on a DB-wax capillary column (30 m × 0.25 mm × 0.25 μm), with the injector port temperature set at 250 °C. The column temperature followed a programmed ramp, as follows: initial temperature of 40 °C held for 5 min, followed by a 5 °C/min increase to 220 °C held for 2.5 min, then a 5 °C/min increase to 250 °C held for 2.5 min. The carrier gas was high-purity He at a flow rate of 1.00 mL/min. The pre-column pressure was 53.5 kPa, with 1 μL injected in splitless mode. The mass spectrometer detector ion source temperature was 230 °C, the transfer line temperature was 260 °C, and the solvent delay was 3 min. The mass scan range was *m*/*z* 29–400, with a scan rate of 1000 u/s.

#### 2.4.6. Determination of Ferric Ion Reducing Power

The phosphate buffer system (PBS, pH 7.4) was prepared by accurately weighing 1.74 g of NaH_2_PO_4_, 2.7 g of Na_2_HPO_4_, and 1.7 g of NaCl by deionized water to 400 mL. A total of 2.5 mL of the PBS working solution was taken in a centrifuge tube, and 0.3 mL of the mulberry fruit wine sample to be tested and 1 mL of 1% K_3_[Fe(CN)_6_] were added sequentially [27]. The mixed system was placed in a 50 °C constant temperature water bath for 20 min and then naturally cooled to 25 ± 2 °C. The mixed system was placed in a 50 °C constant temperature water bath for 20 min and then naturally cooled to 25 ± 2 °C. Protein precipitation was carried out by adding 1 mL of 10% C_2_HCl_3_O_2_, and the supernatant was taken as 2.5 mL after standing for 1 min. Then, 0.5 mL of 0.1% FeCl_3_ solution and 2.5 mL of ultrapure water were injected into the cuvette in sequence, vortexed, and mixed; then, the color was developed after 5 min of standing, and the absorbance value was measured at the wavelength of 700 nm [28].

#### 2.4.7. Determination of Inhibition Effect on Cancer Cell Proliferation

Cell culture: 10% FBS DMEM medium was prepared, inoculated with HepG2 and HT29 cells, and cultured at 37 °C and 5% CO_2_ for 48 h [29]. The MTT method was used to determine the inhibitory effect; 0.25% trypsin digested the cells to 1 × 10^4^ cells/mL, 96-well plates were inoculated with the lyophilized powder of the mulberry fruit wine solution for 24 h, MTT was added, and the sample was then cultured for another 4 h [29]. The culture medium was removed, DMSO was added and shaken for 10 min, the absorbance was measured at 490 nm, and the inhibition rate was calculated [30]. For HepG2 cells, the positive control group used an anticancer drug (5-FU), and for HT29 cells, the positive control group used an anticancer drug (Mat) [31]. A total of 100 µL of the corresponding treatment solution was added to each well, and at least 3 replicate wells were set up for each concentration/group. The incubation was continued for 24 h. It was calculated according to Equation (4).

(4) $$\mathrm{Inhibition}\;\mathrm{rate}\;(\%)=[1-\frac{\mathrm{OD}\;\mathrm{value}\;\mathrm{of}\;\mathrm{experimental}\;\mathrm{group}}{\mathrm{Control}\;\mathrm{group}\;\mathrm{OD}}]\times 100\%$$

with blank correction using DMSO-only wells.

The survival rate of HepG2 and HT29 cells was calculated according to the following formula: cell survival rate (%) = [(OD_sample_ − OD_solvent_)/(OD_control_ − OD_solvent_)] × 100. A four-parameter logistic model was used to fit a dose–effect curve: Y = Bottom + (Top − Bottom)/[1 + 10 *^{(LogIC50 − X) × HillSlope}^], where Y: cell survival (%); X: log_10_ (drug concentration); Top/Bottom: asymptote above/below the curve (constraints: 0 ≤ Bottom ≤ 100, 70 ≤ Top ≤ 100); and HillSlope: slope parameter of the curve. IC_50_ values and their 95% confidence intervals were computed by nonlinear least squares regression, and the model goodness-of-fit determined by coefficient of determination (R^2^ > 0.99) was verified.

#### 2.4.8. Determination of Antimicrobial Activity

The mulberry fruit wines selected for this segment of the antimicrobial activity assay were those fermented for 15 days at an initial sugar level of 26 °Brix and a temperature of 18 °C. The fermentation was carried out at a temperature of 18 °C for 15 days.

A total of 21 g of the MHB medium and 37 g of the BHI medium were weighed accurately, 900 mL of distilled water was added and stirred thoroughly until completely dissolved, and the volume was fixed to 1000 mL and then uniformly dispensed into centrifuge tubes [32]. The centrifuge tubes were sterilized at 121 °C for 15 min and then cooled down and transferred to a refrigerated environment for subsequent experiments. Under aseptic conditions, 20 μL of an overnight culture (approximately 1 × 10^8^ CFU/mL) of E.coli, S.aureus, and S. mutans was inoculated individually into 2 mL of the respective culture medium (MHB for *E. coli* and *S. aureus*; BHI for *S. mutans*), and incubated at a constant temperature for 24 h [33].The strains and culture conditions in the experiment are shown in Table 2.

Mulberry fruit wine was dissolved with an equal volume of DMSO, and antibacterial testing was performed in a sterile room. In this experiment, the starting test concentration of mulberry fruit wine was 100 mg/mL (50% *v*/*v*). Minimum inhibitory concentration (MIC) was determined using the micro broth dilution method in 96-well plates, where the concentration of each bacterial solution after activation was adjusted to approximately 1 × 10^6^ CFU/mL using sterile saline or the corresponding medium under aseptic conditions. Then, 96-well plates were added with 100 μL of culture solution, absorbance was measured at 650 nm by the double dilution method, and the measurements were repeated after incubation at 37 °C for 24 h. The inhibition rate was calculated (Equation (5)), and the minimum inhibitory concentration (MIC) value (μg/mL) was determined [34]. Minimum inhibitory concentration (MIC) is determined by the OD_650_ value, OD_650_ ≤ blank control OD_650_ + 0.05 or less than 10% of the growth control), which is the lowest Mulberry Fruit Wine sample concentration (mg/mL or μg/mL) that can completely inhibit the visible growth of bacteria. The MIC value of each sample for each bacterium should be determined independently, and the experiment should be repeated at least three times.

(5) $$\mathrm{Inhibition}\;\mathrm{rate}\;(\%)=\frac{1-(A-D)}{B-C}\times 100\%$$

where A and B are the absorbance values measured after 20 h of incubation in the experimental and control groups; C and D are the absorbance values measured before 20 h of incubation in the control and experimental groups.

#### 2.4.9. Chromatographic Conditions

After filtration of mulberry fruit wine, 5 mL was placed in a 15 mL headspace flask, and 20 μL of 50 mg/L C_6_H_10_O, 1 g NaCl, and a magnetic rotor were added. After it was filtered by a 0.45 μm membrane, the extract was stirred and adsorbed at 40 °C for 30 min for activation. The extraction head was inserted into the headspace vial, ensuring that it was kept at a distance of about 1 cm from the liquid surface [35]. After adsorption, the extraction head was inserted into the gas chromatography injection port and thermally resolved at 250 °C for 5 min.

##### Mass Spectrometry–Chromatography Conditions

A DB-FFAP polar capillary column (30 m × 0.25 mm × 0.25 μm) was used for the separation of substances, with high-purity helium (≥99.999%) as the carrier gas, and the flow rate was controlled at 1 mL/min. The injection system was used in the manual injection mode, with an injection volume of 1 μL and the non-split flow program enabled. The temperature gradient of the chromatographic column was set as follows: initial 40 °C for 5 min, followed by a programmed temperature increase to 240 °C at a rate of 10 °C/min and maintained for 2 min [16]. The parameters of the mass spectrometry detection system were set as follows: transmission line temperature of 280 °C, ionization source temperature of 230 °C, electron bombardment ionization (EI) mode, electron energy of 70 eV, and mass scanning range of 20–350 amu.

#### 2.4.10. Sensory Evaluation

Twelve screened tasters (consisting of six males and six females, requiring experience in food sensory evaluation, no sensory deficiencies, and an age range of 25–40 years old) were invited to evaluate the samples independently and blindly in a standard sensory laboratory (separate compartments, no intrusive odors, temperature of 22 ± 1 °C, and humidity of 60%), and the criteria for sensory evaluation of the mulberry fruit wines are shown in Table 3.

#### 2.4.11. Quantitative Description of Sensory Evaluation

Quantitative descriptive analysis (QDA) was employed to evaluate the sensory characteristics of rice wine samples. The sensory panel comprised 10 evaluators (5 males and 5 females aged 25–30) with regular experience in the sensory analysis of alcoholic beverages. All participants completed a two-week sensory training course focused on fruit wine aroma profiles. Six sensory descriptors were identified, with definitions and reference points for each shown in Table 4. During sensory analysis, the flavor intensity of the samples ranged from 1 (not detectable) to 15 (extremely intense). Results are presented as averages.

#### 2.4.12. Statistical Analysis

SPSS 22.0 software was used for statistical analysis. Results are expressed as mean ± standard deviation (SD) of three biological replicates (*n* = 3). The analysis of variance and means comparison by Tukey’s test was performed to determine significant differences at *p* ≤ 0.05. Response surface optimization experiments were designed and analyzed using Design-Expert 13.0 software. Principal component analysis was performed, and heatmaps were generated using GraphPad Prism 10.0. Radar charts were created using CNSknowall online charting tools.

## 3. Results and Discussion

### 3.1. One-Way Test to Investigate the Optimal Fermentation Conditions of Mulberry Fruit Wine

**Figure 1:**
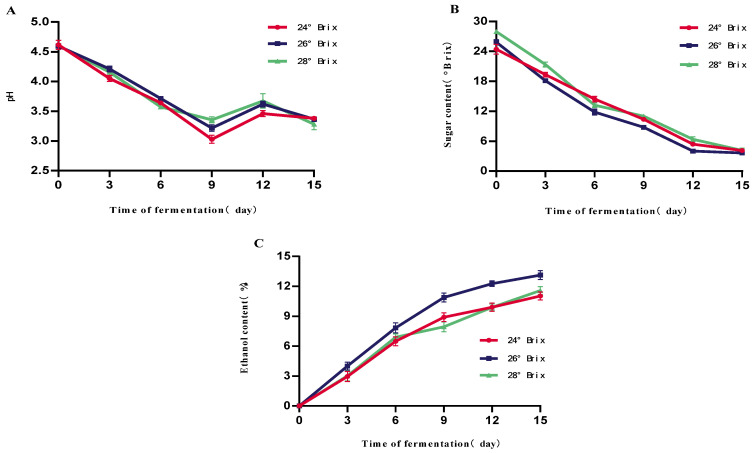
Effect of different initial sugar levels on the fermentation of mulberry fruit wine. (**A**) Changes in mulberry wine pH at different initial fermentation sugar levels (**B**) Changes in mulberry wine sugar content at different initial fermentation sugar levels (**C**) Changes in mulberry wine alcohol content at different initial fermentation sugar levels (Data represent mean ± SD of three independent fermentation batches).

As shown in Figure 1A, the initial pH of the three groups of samples was maintained at about 4.60, and with the advancement of the fermentation process, the pH value showed a significant decreasing trend, reaching the lowest points on the 9th day of fermentation at 3.03, 3.22, and 3.35, respectively. This is because yeast produces a variety of organic acids (e.g., succinic acid, lactic acid, acetic acid, etc.) during the fermentation process, which causes the pH of the fermentation broth to drop. Later on, the subsequent slight increase in pH after day 9 could be attributed to the consumption of organic acids by yeast as carbon sources in the later stages of fermentation and/or the esterification of acids with ethanol to form neutral flavor esters. As shown in Figure 1B, with the prolongation of the fermentation time, the sugar level of the three groups of samples gradually decreased, and the sugar levels decreased slowly from the 9th day to the 15th day, which was due to the fermentation of the wine yeast using glycogen as the substrate to produce ethanol as a substrate for fermentation to produce ethanol. On the 15th day of fermentation, there was no significant difference between the three groups of samples in terms of brix, which were 4.26 °Brix, 3.64 °Brix, and 4.02 °Brix, respectively. This phenomenon is due to fermentation by brewer’s yeast using glycogen as a substrate to produce ethanol. As the sugar decreases significantly, yeast activity decreases, leading to a decrease in ethanol production capacity. The alcohol content of the three groups of samples showed a significant trend in increase with the fermentation time as shown in Figure 1C, and the alcohol content of the three groups of samples was the highest on the 15th day of fermentation, with the highest alcohol contents of 10.5%vol, 12.5%vol, and 13.5%vol, respectively. The alcohol contents of the samples with an initial brix of 26 °Brix were significantly higher than the samples with the same initial brix. The 26 °Brix sample was significantly higher than the other two groups. This is mainly attributed to its higher initial fermentable sugar content providing the yeast with a more adequate substrate, supporting a longer period of effective ethanol synthesis, and ultimately leading to higher ethanol accumulation. It should be noted that °Brix measurements, obtained via refractometer, are influenced by the presence of ethanol and thus represent apparent soluble solids rather than true sugar concentration, particularly in the later stages of fermentation. This may lead to an overestimation of the residual sugar content.

The sensory evaluation panel conducted a comprehensive evaluation of the three groups of samples, which is shown in Table 5. Mulberry fruit wines with an initial brix of 26 °Brix received significantly highest scores (*p* < 0.05) for color (6.10 ± 0.84 ^a^), aroma (5.60 ± 0.97 ^a^), flavor (5.20 ± 0.74 ^a^), and overall evaluation (5.90 ± 0.82 ^a^). This indicates that the fruit wines from this treatment group were visually more vibrant, translucent, and purplish-red in color, with more intense and harmonious fruity and alcoholic aromas; on the palate, they exhibited better body, fruity harmony, and sweet–sour balance characteristics, and they exhibited more pronounced flavors and layers of characteristic mulberry flavors.

**Figure 2:**
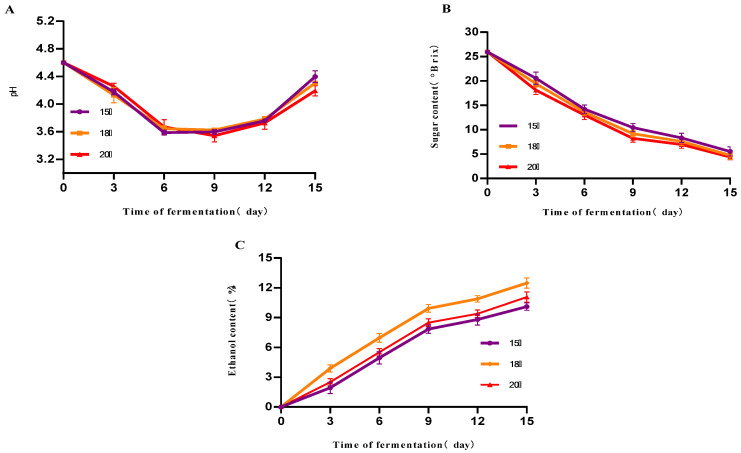
The effect of different temperatures on the fermentation of mulberry fruit wine. (**A**) Changes in mulberry wine pH at different initial fermentation temperatures (**B**) Changes in mulberry wine sugar content at different initial fermentation temperatures (**C**) Changes in mulberry wine alcohol content at different initial fermentation temperatures (Data represent mean ± SD of three independent fermentation batches).

As can be seen from Figure 2A, the initial pH of the three groups of samples was about 4.60 at the different fermentation temperatures of 15 °C, 18 °C and 20 °C. The pH decreased gradually with time during the first 9 days of fermentation. An increasing trend was observed after the 9th day. On the 15th day of fermentation, the pH was 4.40, 4.31 and 4.22, respectively, with no significant difference. From Figure 2B, it can be seen that the sugar levels of the three groups of samples showed a significant decreasing trend with the increase in time, and the lowest sugar levels were found on the 15th day of fermentation, which were 5.51 °Brix, 4.3 °Brix, 4.77 °Brix, respectively, and there was no significant difference. From Figure 2C, it can be seen that the alcohol content of the three groups of samples showed an increasing trend with time, and the increasing trend was influenced in the first 9 days of fermentation. The alcohol content of the sample with a fermentation temperature of 18 °C was 12.5% vol on the 15th day of fermentation, which was significantly higher than that of the other two groups, and this alcohol value was achieved when the residual sugar was reduced to a lower level and the alcohol growth had stabilized, reflecting the higher efficiency of sugar alcohol conversion and the more thorough substrate utilization by the yeast at this temperature. The optimal temperature (18 °C) balances yeast growth, activity, and tolerance to maximize ethanol yield.

As shown in Table 6, the mulberry fruit wines fermented at 18 °C obtained the highest scores (*p* < 0.05) in flavor (5.80 ± 0.71 ^a^) and overall evaluation (5.60 ± 0.52 ^a^), and also maintained a high level in color (6.20 ± 0.48 ^a^) and aroma (5.40 ± 0.47 ^a^). This indicates that the fruit wines obtained at this fermentation temperature were not only attractive in color and aroma but also had a mellow and harmonious taste, with moderate sweetness and sourness, prominent and rich mulberry flavor characteristics, and optimal overall organoleptic quality.

**Figure 3:**
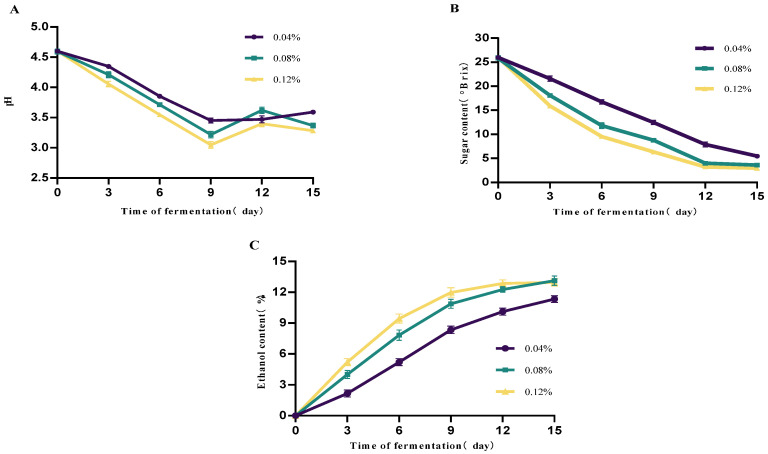
Effect of different yeast dosages on mulberry wine fermentation. (**A**) Changes in mulberry wine pH at different initial yeast additions (**B**) Changes in mulberry wine sugar content at different initial yeast additions (**C**) Changes in mulberry wine alcohol content at different initial yeast additions (Data represent mean ± SD of three independent fermentation batches.).

The effect of the yeast inoculation rate on mulberry fruit wine is shown in Figure 3. The results indicate that inoculation rate significantly influences fermentation kinetic parameters. Higher inoculation rates (≥0.08%) markedly shorten the fermentation lag phase, accelerating sugar consumption and ethanol production. The 0.12% inoculum group exhibited the fastest fermentation rate, with residual sugar nearly depleted by day 9. The 0.08% group also demonstrated good fermentation efficiency, achieving an alcohol content close to that of the high-inoculum group. By contrast, the 0.04% inoculum group fermented slowly, with an extended cycle, higher residual sugar at the end, and significantly lower alcohol content. pH exhibited a decreasing trend throughout fermentation, with the high-inoculation group showing a more rapid pH decline. This was attributed to the yeast metabolism of sugars producing acidic compounds such as carbon dioxide and organic acids (e.g., succinic acid, acetic acid), indicating stronger metabolic activity.

In summary, under the experimental conditions of this study, a 0.08% inoculum level achieved a balance between fermentation efficiency and alcohol yield, making it a suitable inoculation rate.

The yeast addition rate significantly influenced the color, aroma, flavor, and overall sensory score of the finished wine (*p* < 0.05) (Table 7). At a 0.08% addition rate, mulberry wine achieved the highest overall sensory score (5.85 ± 0.28), significantly outperforming the other two groups (*p* < 0.05). It also demonstrated optimal performance in color (6.31 ± 0.97) and flavor (5.39 ± 0.23). Although the 0.12% addition group outperformed the 0.04% group in some indicators, its overall score (5.20 ± 0.65) was significantly lower than that of the 0.08% group, indicating that excessive inoculation may inhibit flavor development. The results suggest that 0.08% is the optimal yeast inoculation level for mulberry wine fermentation.

### 3.2. Response Surface Design Experiment Plan and Results Analysis

Based on the factor-level table in Table 1 for the response surface experiment, a three-factor, three-level experimental design comprising 17 groups was established. The experimental results are presented in Table 8.

Analysis of the response surface design and results indicates that the interactions among the three factors—initial sugar content, fermentation temperature, and yeast addition rate—significantly influence the sensory quality of mulberry wine. The results indicate that under moderate sugar content (approximately 26 °Brix) and an optimal fermentation temperature (17.5 °C), the highest sensory score was achieved at a yeast addition rate of 0.08%. Scores remained consistently above 5.8 across multiple replicate trials, confirming this region as a stable peak zone for sensory response. Significant interactions existed between sugar content and temperature. For instance, the combination of lower sugar content (24 °Brix) and higher temperature (20 °C) (Trial 3) yielded a score of 5.15, while the same temperature applied to higher sugar content (28 °Brix) (Trial 4) further increased the score to 5.20. At a yeast addition rate of 0.12%, pairing with higher temperature (20 °C) and medium sugar content (26 °Brix) yielded the highest score (5.40, Test 12). However, at the same temperature, reducing yeast to 0.04% while maintaining sugar content resulted in a noticeable decline in score (5.30, Test 10), indicating that higher yeast addition at elevated temperatures aids flavor development. Considering all interaction effects, the optimal process parameters for mulberry wine production are recommended as follows: initial sugar content of 26–28 °Brix, fermentation temperature of 19–20 °C, and yeast addition rate of 0.08–0.12%. This range balances fermentation efficiency with sensory flavor quality.

This study utilized Design-Expert 13.0 software to analyze the Box–Behnken Design (BBD) response surface data from Table 8, which featured three factors at three levels. Sensory scores were employed as the response variable, and a second-order response surface regression model was applied for regression fitting. Through analytical calculations, the regression equation for sensory scores was successfully derived as follows: Y = 5.90 + 0.0250 * A + 0.2625 * B + 0.1250 * C + 0.0000 * AB + 0.0000 * AC − 0.0250 * BC − 0.6260 * A^2^ − 0.4010 * B^2^ − 0.3760 * C^2^.

The analysis of variance for the sensory evaluation regression model of mulberry fruit wine is shown in Table 9 and Table 10. The results indicate that the overall model is highly significant (*p* < 0.0001), demonstrating that the constructed model possesses high statistical significance. Among the primary terms, fermentation temperature (B) and the yeast inoculation level (C) exerted highly significant effects on sensory scores (*p* < 0.0001), while initial sugar content (A) showed no significant influence (*p* = 0.2469). The quadratic terms A^2^, B^2^, and C^2^ were all highly significant (*p* < 0.0001), indicating a clear nonlinear relationship between each factor and sensory score. Among the interaction terms, only BC (fermentation temperature and yeast addition rate) showed a significant effect (*p* = 0.0004), while the AB and AC interactions were not significant. The model exhibited high goodness-of-fit (R^2^ = 0.9952), low coefficient of variation (C.V. % = 1.08), and an Adeq Precision of 37.2596—significantly greater than 4. This indicates excellent predictive accuracy and reliability, making the model suitable for optimizing the sensory quality of mulberry wine.

The study conducted pairwise interaction effect analysis on initial sugar content (A), fermentation temperature (B), and the yeast inoculation rate (C), plotting response surface diagrams. Generally, steeper response surfaces with denser contour lines indicate more pronounced factor effects; contour lines approaching elliptical shapes signify stronger interactions between factors. This study conducted pairwise interaction analyses for initial sugar content (A), fermentation temperature (B), and the yeast inoculation rate (C), generating response surface plots. These plots provide clear insights into the interactions among factors and help determine optimal parameter settings. Response surfaces and contour plots visually illustrate how factor interactions influence response values. When any variable is held at its central level while the other two vary within the optimization range, the combined effects of all factors can be demonstrated through contour plots and response surfaces. Generally, steeper response surfaces and denser contour lines indicate more pronounced factor effects; the closer the contour shape approaches an ellipse, the stronger the interaction between the two factors.

**Figure 4:**
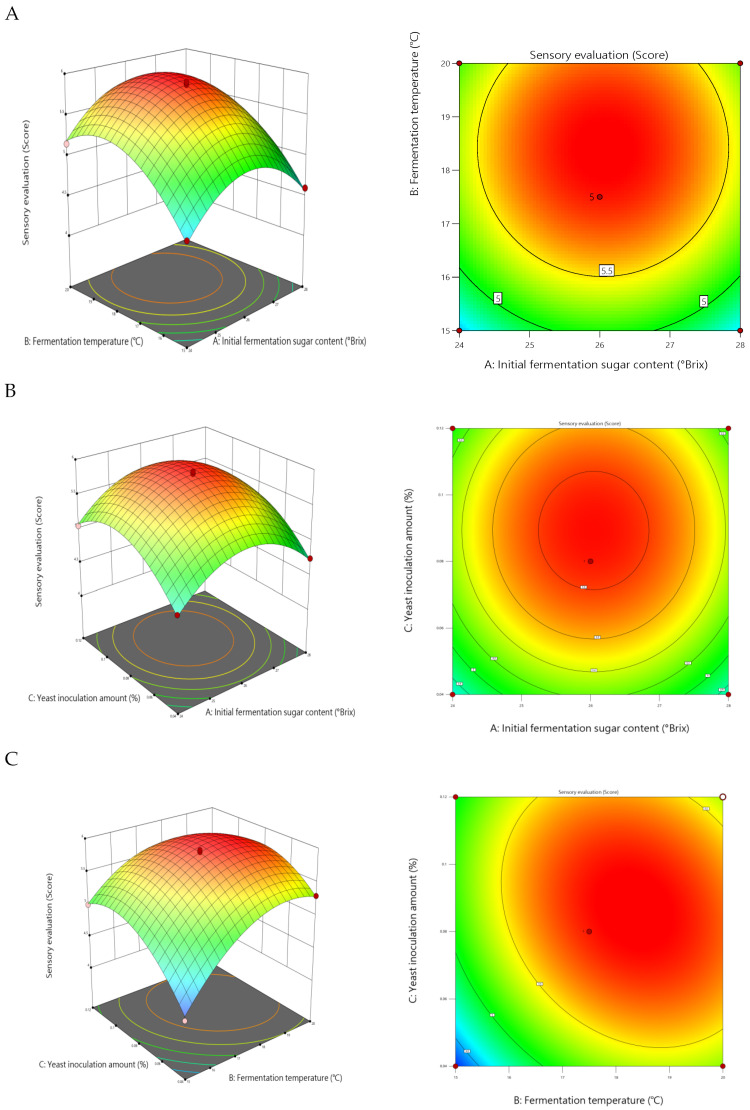
Response surface plots showing the effects of interactions between factors on sensory scores: (**A**) initial sugar content versus fermentation temperature, (**B**) yeast inoculum versus initial sugar content, and (**C**) yeast inoculum versus fermentation temperature.

As shown in Figure 4, the interaction effect between fermentation temperature (B) and the yeast addition rate (C) (BC) reached a highly significant level (*p* = 0.0004). The response surface curve exhibited steep gradients with a significant rate of change. Particularly at low yeast addition levels (C ≈ 0.04%), the rate of decline in sensory scores increased markedly with the rise in temperature. This indicates that temperature variations more readily induce sensory quality fluctuations when yeast activity is constrained. The contour lines formed distinct elliptical shapes, further confirming the strong interaction between the two factors. Within the fermentation temperature range of 17–19 °C and yeast addition levels of 0.10–0.12%, predicted sensory scores exceeded 5.7, defining the premium quality zone for this factor combination.

By contrast, the interactions between initial sugar content and fermentation temperature (AB) and the yeast inoculation rate (AC) were not statistically significant (*p* > 0.05). The response surface for the AB interaction exhibited a relatively flat profile, with circular and sparsely distributed contour lines, indicating that temperature’s influence on sensory scores followed a consistent pattern across different sugar content levels. The AC interaction also exhibited a similarly weak synergistic effect. Although both interactions were statistically insignificant, combining them with the single-factor effects revealed that sugar content within the 24–28 °Brix range, when paired with medium-to-high yeast addition levels (0.10–0.12%), helped maintain higher sensory acceptability.

In summary, the interaction between fermentation temperature and the yeast inoculation rate represents the most significant synergistic factor influencing the sensory quality of mulberry wine. Maximizing sensory scores can be achieved by controlling the temperature between 17 and 19 °C, maintaining a yeast inoculation rate of 0.10–0.12%, and ensuring an initial sugar content of 26–28 °Brix. This result clearly identifies the key pathway for multi-parameter synergistic optimization, providing theoretical basis and data support for precise process control in mulberry wine production.

To maximize the predicted values of alcohol content and sensory scores for the response, thereby achieving optimal results, an effective regression model was employed for condition optimization. The response surface software prediction module was used to determine the optimal solution. The objective function and mathematical model for optimization are as follows:

$$\begin{array}{l} Y(\mathrm{Sensory}\;\mathrm{evaluation}\;\mathrm{Score})(\mathrm{MAX})\\ 24\leq A\leq 28\\ 15\leq B\leq 20\\ 0.08\leq C\leq 0.12 \end{array}$$

The actual values of each factor corresponding to optimal conditions are initial sugar content (A): 25.058 °Brix; fermentation temperature (B): 17.976 °C; and the yeast addition rate (C): 0.087836%. The model predicted a sensory score of 5.79628 points. Considering practical conditions and operability, parameters were set as follows: initial sugar content (A): 25 °Brix; fermentation temperature (B): 18 °C; and yeast addition rate (C): 0.08%. Three replicate experiments under these conditions yielded a validated average sensory score of 5.83 points. The relative error between predicted and measured values was less than 5%. Detailed experimental results are presented in Table 11.

### 3.3. Volatile Aroma Compounds in Mulberry Fruit Wine and Mulberry Juice Under Optimal Fermentation Conditions

**Figure 5:**
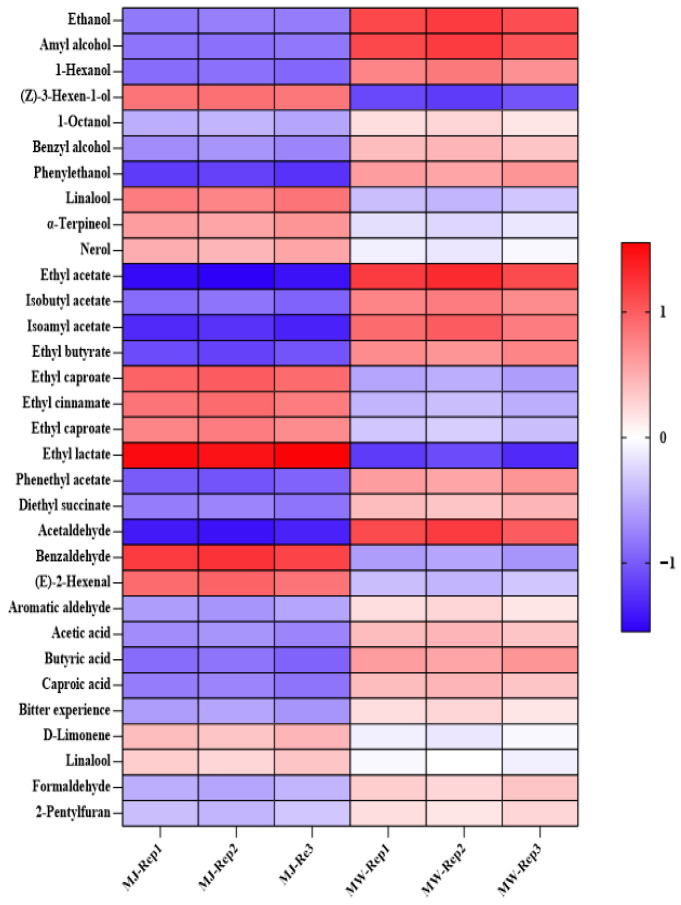
Heatmap of volatile aroma compound content in mulberry fruit wine and mulberry juice under optimal fermentation conditions.

To clarify the fundamental influence of fermentation processes on the flavor composition of mulberry products, we prepared mulberry wine (MW) under optimized conditions (initial sugar content: 25 °Brix; fermentation temperature: 18 °C; yeast inoculation rate: 0.08%) and compared it with unfermented mulberry juice (MJ) as a control. Volatile aroma compounds were identified and quantified using GC-MS. Each group included three biological replicates, with results expressed as relative content (%). See Table 12 and Figure 5.

Alcohols form the essential framework of a wine’s flavor profile. As shown in Table 12, the fermentation process significantly alters the composition and content of alcoholic compounds. Ethanol, the primary product of alcoholic fermentation, surged dramatically from an average of 0.88% in the juice to 8.98% in the wine, confirming the thorough metabolic activity of the yeast. Higher alcohols also increased several to dozens of times over. At low concentrations, these higher alcohols impart a mellow mouthfeel to the wine, but excessive amounts may produce off-flavors. Notably, the content of (Z)-3-hexen-1-ol, which contributes a grassy aroma, slightly decreased after fermentation, while the content of phenethyl alcohol, which imparts a rose-like fragrance, significantly increased (from 0.065% to 0.352%), positively contributing to the wine’s aroma enhancement. Esters are the primary source of fruity and sweet aromas in fruit wines, and their changes represent the most significant flavor transformation during fermentation. Following fermentation, the majority of the esters exhibited orders-of-magnitude increases in concentration. Ethyl acetate (exhibiting pineapple and fruity notes) rose from 0.015% to 1.852%, representing a 123-fold increase. Isoamyl acetate (exhibiting banana and pear notes) surged from 0.008% to 0.845%, exceeding a hundredfold increase. Other esters like ethyl butyrate and ethyl caprylate also showed significant increases. The synergistic effect of these esters collectively formed the rich, harmonious, and complex fruit aroma of mulberry wine. Conversely, ethyl lactate—the most abundant ester in the juice—was largely consumed during fermentation, decreasing from 1.852% to 0.185%. This suggests it likely served as a substrate for yeast secondary metabolism. Among aldehydes, acetaldehyde—a fermentation intermediate—showed substantial increase (from 0.002% to 0.865%), playing a crucial role in forming the wine’s “winey” aroma. Conversely, benzaldehyde (bitter almond aroma), which was relatively high in the juice, decreased post-fermentation (from 0.235% to 0.125%). Organic acids constituted the primary acidity of the wine. Short-chain fatty acids like acetic acid and butyric acid moderately increased post-fermentation, enriching the wine’s acidity profile. Additionally, furan compounds (e.g., 2-pentylfuran) were newly detected after fermentation. These compounds typically impart fruity and nutty aromas, adding complexity to the wine’s bouquet.

**Figure 6:**
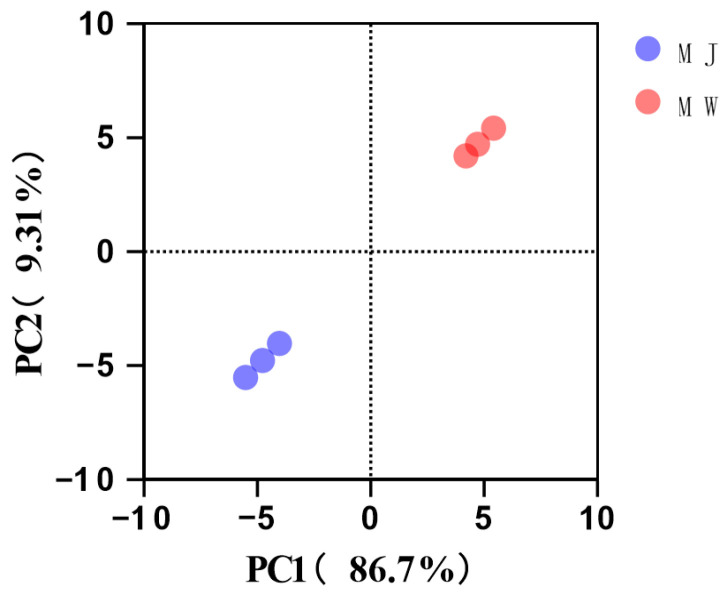
Principal component analysis (PCA) of volatile components in mulberry fruit wine and mulberry juice under optimal fermentation conditions.

To comprehensively evaluate the impact of fermentation on aroma profiles, principal component analysis (PCA) was performed on the volatile component data of all samples. As shown in Figure 6, the cumulative variance contribution of PC1 and PC2 reached 96.01% (PC1: 86.7%, PC2: 9.31%), fully reflecting the original data information. The score plot clearly shows that the three replicates of mulberry juice (MJ) and the three replicates of mulberry wine (MW) cluster on opposite sides of the PC1 axis, forming two distinct and distant clusters. This indicates extremely significant differences in the overall aroma composition between juice and wine, with good biological repeatability within each group. The PC1 axis serves as the decisive factor distinguishing fermented from unfermented samples. Its high contribution rate of 86.7% corroborates the dramatic changes in the aforementioned volatile compounds.

### 3.4. Sensory Analysis of Optimized Mulberry Wine (QDA)

Sensory characteristics are key factors in gaining consumer acceptance for fermented beverages. We conducted a comprehensive descriptive sensory analysis of mulberry wine produced via an optimized fermentation process and unfermented mulberry juice (three biological replicates each) to characterize their key sensory attributes.

**Figure 7:**
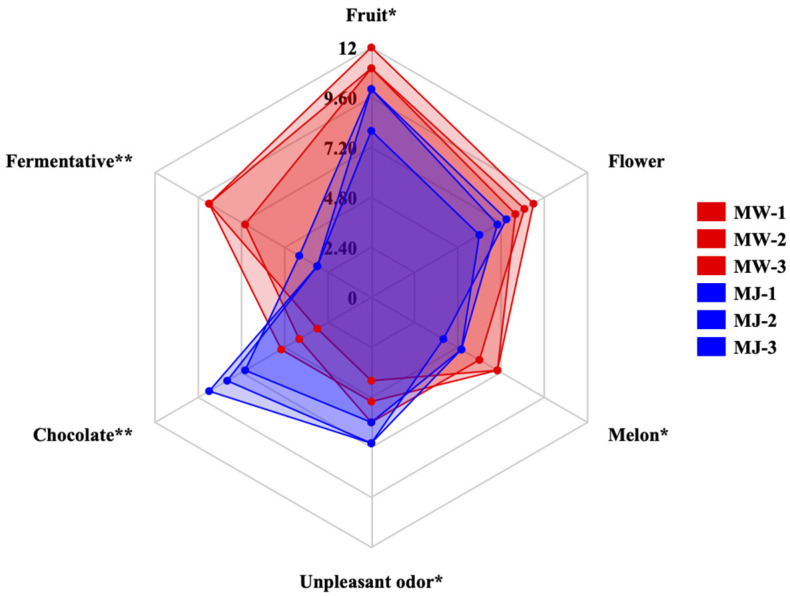
Comparison of aroma profiles between mulberry wine and mulberry juice samples under optimal fermentation conditions (** *p* < 0.05, ** *p* < 0.01).

As shown in Figure 7, mulberry fruit wine can be described using six aroma attributes: fruity, floral, melon, fermented, chocolate, and off-flavor. Furthermore, the intensity of these aroma attributes varies among different samples. One-way ANOVA revealed extremely significant differences (*p* < 0.01) in fermentative and chocolate attributes among samples, with significant differences (*p* < 0.05) in fruit, melon, and off-flavor attributes. Mulberry wine (MW) exhibited significantly higher intensity in fruit and fermentative attributes compared to mulberry juice (MJ). This result strongly correlates with the significant increase in ester compounds (e.g., ethyl acetate, amyl acetate) detected by GC-MS, confirming that yeast fermentation metabolism effectively endows mulberry wine with richer, more complex composite fruit aromas. Unpleasant odors (e.g., grassy, raw) originally present in mulberry juice were significantly reduced in intensity after fermentation. This indicates that the fermentation process effectively eliminated certain undesirable flavor precursors in the juice. Additionally, evaluators detected faint chocolate notes in some samples, potentially linked to flavor compounds like pyrazines and furans formed during fermentation or aging, adding a unique complexity to the product.

The scores for mulberry wine across various aroma attributes showed concentrated distribution across three biological replicates (MW-1, MW-2, MW-3), indicating excellent flavor stability and high reproducibility when produced under optimal process parameters. By contrast, the sample points for mulberry juice (MJ-1, MJ-2, MJ-3) exhibited relatively dispersed distribution, reflecting the inherent instability of its natural matrix flavor.

### 3.5. Antioxidant Activity of Mulberry Fruit Wine and Mulberry Juice Under Optimal Fermentation Conditions

**Figure 8:**
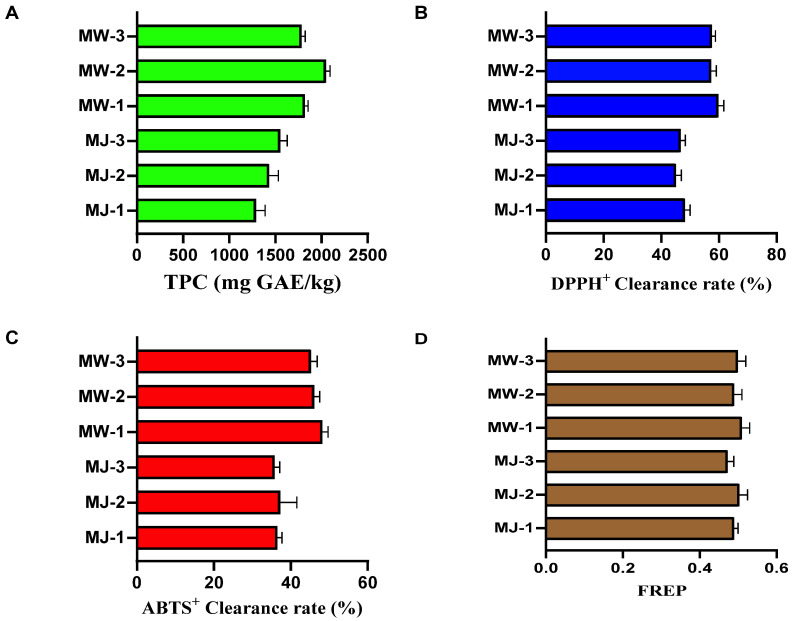
Antioxidant properties of mulberry fruit wine and mulberry juice under optimal fermentation conditions ((**A**) total phenolic content; (**B**) DPPH^+^ radical scavenging activity; (**C**). ABTS^+^ radical scavenging activity; (**D**) ferric-ion-reducing antioxidant power).

To investigate the effects of fermentation processes on the functional components and antioxidant capacity of mulberry products, this study systematically evaluated the antioxidant activity of mulberry wine (MW) prepared under optimal conditions and its raw material, mulberry juice (MJ). Assessments included total phenolic content, DPPH radical scavenging rate, ABTS^+^ radical scavenging rate, and ferrous reduction ability (FRAP). Each sample underwent three biological replicates. The results are shown in Figure 8.

As shown in Figure 8A, after fermentation, the total phenolic content of mulberry wine was significantly higher than that of mulberry juice. The mean total phenolic content in the juice group (MJ) was 1454.67 ± 132.41 mg GAE/kg, while that in the wine group (MW) reached 1901.33 ± 130.55 mg GAE/kg, representing an increase of approximately 30.7%. These results indicate that the yeast fermentation process significantly promoted the extraction and conversion of phenolic compounds in mulberry fruits. This effect is primarily attributed to ethanol, acting as an organic solvent, enhancing the extraction efficiency of bound phenolic compounds within cell walls. Figure 8B,C show mutually corroborating results from two radical scavenging assays, collectively demonstrating that the in vitro antioxidant activity of mulberry wine substantially increased after fermentation. The FRAP assay in Figure 8D assessed antioxidant potential by measuring the sample’s ability to reduce Fe^3+^ to Fe^2+^. The FRAP value of mulberry wine was slightly higher than that of mulberry juice (mean 0.49 ± 0.02). Although the absolute increase was modest, the trend was consistent and statistically significant (*p* < 0.05). Alcoholic fermentation significantly improved the antioxidant activity of mulberries. Specifically, total phenolic content increased by approximately 30.7%, DPPH and ABTS radical scavenging rates both rose by about 25–30%, and FRAP reduction capacity also strengthened. These findings reveal the added value of mulberry wine over mulberry juice at the functional activity level. This not only supports its flavor acceptability but also provides scientific basis for developing it as a functional food with potential health benefits.

### 3.6. Determination of the Inhibitory Effect of Freeze-Dried Powder Solution of Mulberry Fruit Wine on the Proliferation of Cancer Cells

As a commonly used technique for the determination of cell proliferation and viability, the MTT assay has an important application in cellular assays. Its mechanism of action lies in the fact that specific dehydrogenases present in the mitochondria of living cells are able to convert MTT into insoluble blue-violet crystalline products, which can be completely dissolved upon contact with dimethyl sulfoxide (DMSO).

**Figure 9:**
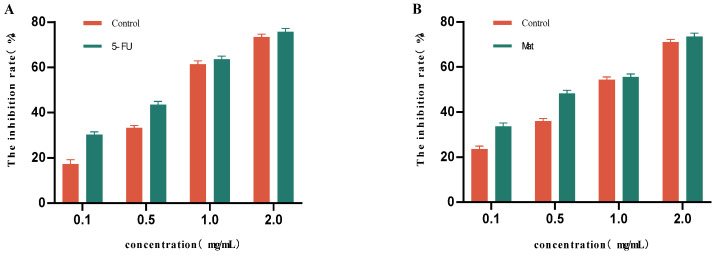
Inhibitory effects of different concentrations of mulberry fruit wine lyophilized powder solutions on HepG2 and HT29 cells. (**A**) Inhibition rate of HepG2 cells as a function of mulberry wine freeze-dried powder solution concentration. (**B**) Inhibition rate of HT29 cells as a function of mulberry wine freeze-dried powder solution concentration (Data represent mean ± SD of three independent fermentation batches).

For this part of the study, we selected freeze-dried powder made from mulberry fruit wine fermented under optimal conditions (initial fermentation sugar content: 25 °Brix; fermentation temperature: 18 °C; yeast addition rate: 0.08%). As can be seen from Figure 9A, different concentrations of the mulberry fruit wine lyophilized powder solution produced certain inhibitory effects on HepG2 cells, the inhibitory effect was not significantly different from that of the control group at the concentrations of 1.0 mg/mL and 2.0 mg/mL, and the inhibition rate of HepG2 cells reached 61.41% and 73.28%, respectively. As can be seen from Figure 9B, the inhibition rate of HT29 cells showed a gradual increase with the increase in the concentration of the mulberry fruit wine lyophilized powder solution. The inhibitory effects at concentrations of 1.0 mg/mL and 2.0 mg/mL were not significantly different from those of the control group, and the inhibition rates of HT29 cells reached 54.32% and 70.96%, respectively.

The results, presented as follows, were analyzed by a four-parameter logistic Sti model: HepG2 cells: IC_50_ = 0.82 mg/mL (95% CI: 0.78–0.86, R^2^ = 0.996); HT29 cells: IC_50_ = 1.05 mg/mL (95% CI: 0.99–1.11, R^2^ = 0.992). The positive control 5-FU had an IC_50_ of 0.75 mg/mL for HepG2 cells, and MAT had an IC_50_ of 0.98 mg/mL for HT29 cells.

Mulberry fruit wine may have potential anticancer effects due to its richness in various phenolic compounds. The mechanism of action of natural active ingredients such as tumor-preventive factors involves accelerating the process of programmed cell death and influencing signaling pathways by regulating the expression of key genes. The inhibitory effects of mulberry fruit wine on other types of cancer cells and their specific pathways of action still need to be explored in depth.

### 3.7. Results of Antimicrobial Properties of Mulberry Fruit Wine

**Figure 10:**
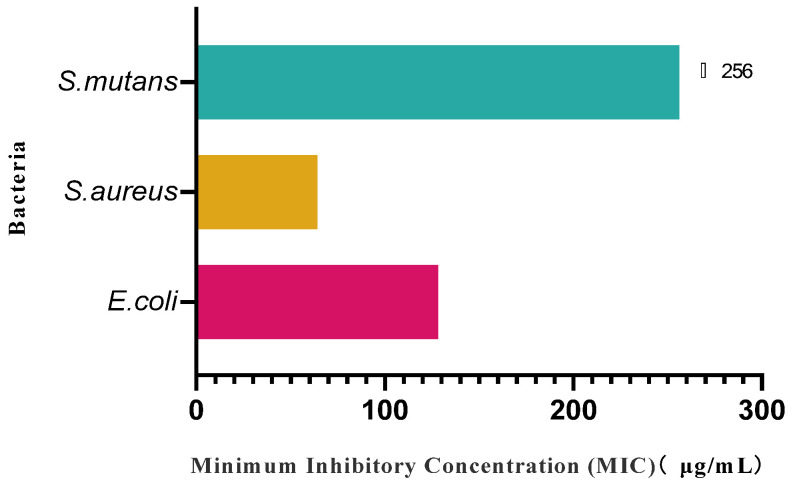
Inhibitory effect of mulberry fruit wine on three pathogenic bacteria. (Data represent mean ± SD of three independent fermentation batches).

As can be seen from Figure 10, mulberry fruit wine showed significant antimicrobial activity against *E. coli* (1682) and *S. aureus* (503), with MIC = 128 μg/mL for *E. coli* and MIC = 64 μg/mL for *S. aureus*, and in the concentration ranges tested in the present experiments (up to and including 256 μg/mL), no significant inhibitory effect (MIC > 256 μg/mL) was observed against *S. mutans* (3289). In the concentration range tested in this experiment (up to 256 μg/mL or 256 μg/mL), mulberry fruit wine did not show a significant inhibitory effect on *S. mutans* (3289) (MIC > 256 μg/mL). The present study demonstrated that mulberry fruit wine has the ability to inhibit both Gram-negative (*E.coli*) and Gram-positive (*S. aureus*) bacteria, and this antimicrobial activity may also be attributed to its rich phenolic compounds, which may act through the mechanism of disrupting the cell membrane structure, inhibiting the activity of key enzymes, or interfering with the metabolic pathways. The lack of a significant inhibitory effect against *S. mutans* may be due to the fact that the bacterium is more tolerant to phenolics or a result of the limited effect of specific active ingredients in mulberry fruit wines on this bacterium. The isolation and characterization of the antibacterial spectrum, mechanism of action, and active ingredients of other microorganisms will be the focus of future research.

### 3.8. Limitations (Key Strategies)

The in vitro anticancer and antimicrobial activities observed are promising and are likely attributed to the rich polyphenolic profile of the wine. However, these findings are preliminary. Further in vivo studies and detailed mechanistic investigations are necessary to validate these effects and elucidate the precise pathways involved.

## 4. Conclusions

This study successfully optimized the fermentation process of mulberry wine through a systematic approach combining single-factor experiments with response surface methodology. The optimal conditions identified are listed as follows: an initial sugar content of 25 °Brix, fermentation temperature of 18 °C, and yeast inoculation rate of 0.08%. Under these conditions, the product exhibited superior sensory quality, richer volatile aroma characteristics, and enhanced antioxidant capacity. Notably, the post-fermentation wine exhibited significantly higher phenolic compound content than unfermented juice, along with enhanced radical scavenging capacity. Furthermore, this mulberry wine study demonstrates, for the first time, the potent dose-dependent anticancer activity of mulberry wine against HepG2 and HT29 cells, alongside selective antibacterial effects against Gram-negative and Gram-positive pathogens. These findings underscore the fact that mulberry wine is not only a delicious fermented beverage but also a functional food with potential health benefits. Further research is needed to elucidate the underlying mechanisms of its bioactivity and validate these effects in vivo.

## Figures and Tables

**Table 1:** Response surface analysis experiment factors and levels.

Factor	Number	Level		
		−1	0	1
Initial Fermentation Sugar Content (°Brix)	A	24	26	28
Fermentation Temperature (°C)	B	15	18	20
Yeast Inoculation Rate (%)	C	0.04	0.08	0.12

**Table 2:** Bacterial strains and culture conditions in the experiment.

Strains	Conditional			
	A (Temperature) °C	B (Culture Medium)	C (Time)/h	Strain Identifier /Source
*E. coli*	37	MHB	24	ATCC 1682
*S.aureus*	37	MHB	24	ATCC 503
*S.mutans*	37	BHI	24	CGMCC 3289

**Table 3:** Mulberry fruit wine sensory rating criteria.

	Score	Evaluation Criteria
Color	7-5	Vibrant, clear, deep fuchsia colors
	4-2	No sediment, weak gloss
	2-1	Turbid and opaque, dull color
Aroma	7-6	Fruity aroma and aging flavor
	6-4	Fruity aroma and clean, unadulterated flavor
	4-2	Weak aroma with yeast-like odor
	1	Noticeable irritating odor
Taste	7-5	Fruity and well-balanced with sweet and sour notes.
	5-2	Thin on the palate, with a slightly bitter undertone and astringency
	2-1	Significant bitterness
Synthesized assessment	7-5	Unique mulberry flavor and richness
	5-2	Mulberry features are unremarkable and poorly layered
	2-1	Lack of mulberry flavor qualities

**Table 4:** Sensory descriptors and definitions for mulberry fruit wine.

Sensory Descriptors	Definition	Reference Sample
Fruit	A delightful, blended sweet and sour aroma	Fresh Mulberry Puree
Flower	A sweet fragrance reminiscent of the scent of flowers such as roses, violets, or lilies	Phenylethanol
Chocolate	A baked aroma reminiscent of dark chocolate or cocoa, with a hint of bittersweetness and richness	Dark chocolate with ≥70% cocoa content
Melon	Sweet and refreshing aroma, reminiscent of ripe cantaloupe or honeydew melon	Fresh Honeydew Melon or Zespri Gold Kiwifruit
Fermentative	The complex bouquet derived from ethanol and other fermentation byproducts, with a pure, pleasant wine aroma	Ethanol
Unpleasant odor	A general term for unpleasant off-flavors, such as acetic acid taste, oxidized taste, musty taste, or excessive yeast flavor	Acetic acid solution

**Table 5:** Effect of different initial sugar levels on the sensory evaluation of mulberry fruit wine.

Initial Brix	Color/Score	Aroma/Score	Flavor/Score	Comprehensive Evaluation/Score
24 °Brix	4.90 ± 1.08 ^b^	4.20 ± 0.86 ^b^	4.60 ± 0.47 ^a^	4.70 ± 0.63 ^b^
26 °Brix	6.10 ± 0.84 ^a^	5.60 ± 0.97 ^a^	5.20 ± 0.74 ^a^	5.90 ± 0.82 ^a^
28 °Brix	5.00 ± 0.48 ^b^	4.30 ± 0.63 ^b^	4.70 ± 0.53 ^a^	4.80 ± 0.57 ^b^

Values are presented as mean ± SD. Different superscript letters indicate significant differences (*p* < 0.05).

**Table 6:** Effect of different fermentation temperatures on the sensory evaluation of mulberry fruit wine.

Fermentation Temperature	Color/Score	Aroma/Score	Flavor/Score	Comprehensive Evaluation/Score
15 °C	4.71 ± 0.87 ^b^	4.38 ± 0.73 ^b^	4.91 ± 0.25 ^a^	4.63 ± 0.39 ^b^
18 °C	5.71 ± 0.65 ^a^	6.05 ± 0.29 ^a^	5.42 ± 0.61 ^a^	5.87 ± 0.77 ^a^
20 °C	4.90 ± 0.49 ^b^	5.39 ± 0.45 ^b^	5.17 ± 0.42 ^a^	5.36 ± 0.62 ^b^

Values are presented as mean ± SD. Different superscript letters indicate significant differences (*p* < 0.05).

**Table 7:** Effect of different yeast dosages on the sensory evaluation of mulberry wine.

Fermentation Temperature	Color/Score	Aroma/Score	Flavor/Score	Comprehensive Evaluation/Score
0.04%	5.20 ± 0.75 ^b^	4.85 ± 0.82 ^b^	4.50 ± 0.68 ^b^	4.80 ± 0.70 ^b^
0.08%	6.31 ± 0.97 ^a^	5.54 ± 0.71 ^b^	5.39 ± 0.23 ^a^	5.85 ± 0.28 ^a^
0.12%	5.80 ± 0.60 ^a^	5.10 ± 0.89 ^b^	4.90 ± 0.55 ^b^	5.20 ± 0.65 ^b^

Values are presented as mean ± SD. Different superscript letters indicate significant differences (*p* < 0.05).

**Table 8:** Experimental plan and results of the response surface design.

Number	Run	Factors			Result
		Initial Sugar Content (A)	Fermentation Temperature (B)	Yeast Addition Rate (C)	Sensory Evaluation
1	16	24	15	0.08	4.55
2	14	28	15	0.08	4.6
3	4	24	20	0.08	5.15
4	9	28	20	0.08	5.2
5	10	24	17.5	0.04	4.7
6	1	28	17.5	0.04	4.75
7	5	24	17.5	0.12	5.05
8	15	28	17.5	0.12	5.1
9	12	26	15	0.04	4.2
10	13	26	20	0.04	5.3
11	17	26	15	0.12	5
12	11	26	20	0.12	5.4
13	3	26	17.5	0.08	5.88
14	7	26	17.5	0.08	5.87
15	8	26	17.5	0.08	5.8
16	2	26	17.5	0.08	5.9
17	6	26	17.5	0.08	5.86

**Table 9:** Analysis of variance for regression models.

Sources	Sensory Evaluation Score					
	Sum of Squares	df	Mean Square	F-Value	*p*-Value	Significant
Model	4.53	9	0.5038	160.82	<0.0001	**
A	0.0050	1	0.0050	1.60	0.2469	
B	0.9113	1	0.9113	290.87	<0.0001	**
C	0.3200	1	0.3200	102.14	<0.0001	**
AB	0.0000	1	0.0000	0.0000	1.0000	
AC	0.0000	1	0.0000	0.0000	1.0000	
BC	0.1225	1	0.1225	39.10	0.0004	
A^2^	1.19	1	1.19	378.95	<0.0001	**
B^2^	0.8755	1	0.8755	279.46	<0.0001	**
C^2^	0.7822	1	0.7822	249.66	<0.0001	**
Residual	0.0219	7	0.0031			
Lack of Fit	0.0162	3	0.0054	3.81	0.1143	
Pure Error	0.0057	4	0.0014			
Cor Total	4.56	16				

** *p* < 0.01.

**Table 10:** Analysis of variance table for response surface model.

Response Value Metric	Mean	Std. Dev	R^2^	C.V.%	Adeq Precision
Sensory Evaluation Score	5.19	0.0560	0.9952	1.08	37.2596

**Table 11:** Validation test results.

Number	Parameters			Sensory Evaluation
	Initial Sugar Content (A)	Fermentation Temperature (B)	Yeast Addition Rate (C)	
1				5.5
2	25	18	0.08%	6.5
3				5.5
Mean				5.83

**Table 12:** Volatile aroma compound content in mulberry fruit wine and mulberry juice under optimal fermentation conditions.

Compound Name	CAS Number	Relative Content (%)					
		Mulberry Juice			Mulberry Wine		
		MJ-1	MJ-2	MJ-3	MW-1	MW-2	MW-3
Ethanol	000064-17-5	0.85	0.92	0.88	8.95	9.32	8.68
Amyl alcohol	000123-51-3	0.032	0.028	0.035	0.418	0.452	0.385
1-Hexanol	000111-27-3	0.015	0.018	0.012	0.125	0.138	0.112
(Z)-3-Hexen-1-ol	000928-96-1	0.008	0.006	0.01	0.005	0.004	0.003
1-Octanol	000111-87-5	0.002	0.003	0.001	0.008	0.009	0.007
Benzyl alcohol	000100-51-6	0.045	0.051	0.039	0.088	0.095	0.081
Phenylethanol	000060-12-8	0.065	0.071	0.059	0.352	0.328	0.375
Linalool	000078-70-6	0.018	0.015	0.021	0.012	0.01	0.014
α-Terpineol	000098-55-5	0.006	0.004	0.008	0.005	0.003	0.007
Nerol	000106-25-2	0.003	0.002	0.004	0.002	0.001	0.003
Ethyl acetate	000141-78-6	0.015	0.012	0.018	1.852	1.965	1.738
Isobutyl acetate	000110-19-0	0.001	0.002	0	0.235	0.248	0.222
Isoamyl acetate	000123-92-2	0.008	0.01	0.006	0.845	0.912	0.778
Ethyl butyrate	000105-54-4	0.005	0.003	0.007	0.328	0.295	0.361
Ethyl caproate	000123-66-0	0.022	0.025	0.019	0.105	0.118	0.092
Ethyl cinnamate	000106-32-1	0.011	0.014	0.008	0.085	0.091	0.079
Ethyl caproate	000110-38-3	0.007	0.009	0.005	0.032	0.035	0.029
Ethyl lactate	000097-64-3	1.852	1.765	1.938	0.185	0.202	0.168
Phenethyl acetate	000103-45-7	0.004	0.002	0.006	0.158	0.142	0.174
Diethyl succinate	000123-25-1	0.009	0.011	0.007	0.095	0.088	0.102
Acetaldehyde	000141-78-6	0.002	0.001	0.003	0.865	0.921	0.809
Benzaldehyde	000110-19-0	0.235	0.248	0.222	0.125	0.138	0.112
(E)-2-Hexenal	000123-92-2	0.015	0.018	0.012	0.005	0.004	0.006
Aromatic aldehyde	000105-54-4	0.003	0.002	0.004	0.008	0.009	0.007
Acetic acid	000110-38-3	0.025	0.03	0.02	0.215	0.238	0.192
Butyric acid	000097-64-3	0.008	0.01	0.006	0.085	0.078	0.092
Caproic acid	000103-45-7	0.012	0.015	0.009	0.045	0.052	0.038
Bitter experience	000123-25-1	0.018	0.021	0.015	0.032	0.035	0.029
D-Limonene	005989-27-5	0.005	0.004	0.006	0.004	0.003	0.005
Linalool	000123-35-3	0.002	0.001	0.003	0.001	0.002	nd
Formaldehyde	000098-01-1	nd.	nd.	nd.	0.035	0.028	0.042
2-Pentylfuran	003777-69-3	0.001	nd.	0.002	0.015	0.012	0.018

nd: Not detected.

## Data Availability

The original contributions presented in the study are included in the article, further inquiries can be directed to the corresponding author.
